# An Optical Fiber Bundle Sensor for Tip Clearance and Tip Timing Measurements in a Turbine Rig

**DOI:** 10.3390/s130607385

**Published:** 2013-06-05

**Authors:** Iker García, Josu Beloki, Joseba Zubia, Gotzon Aldabaldetreku, María Asunción Illarramendi, Felipe Jiménez

**Affiliations:** 1 Department of Communications Engineering, University of the Basque Country, Alda. Urquijo s/n Bilbao 48013, Spain; E-Mails: joseba.zubia@ehu.es (J.Z.); gotzon.aldabaldetreku@ehu.es (G.A.); 2 CTA, Centro de Tecnologías Aeronáuticas, Parque tecnológico de Bizkaia, Edif. 303, Zamudio 48170, Spain; E-Mail: josu.beloki@ctabef.com; 3 Department of Applied Physics I, University of the Basque Country, Alda. Urquijo s/n Bilbao 48013, Spain; E-Mail: ma.illarramendi@ehu.es; 4 Department of Applied Mathematics, University of the Basque Country, Alda. Urquijo s/n Bilbao 48013, Spain; E-Mail: felipe.jimenez@ehu.es

**Keywords:** optical fiber bundle, real operating conditions measurement, tip clearance, tip timing, turbine

## Abstract

When it comes to measuring blade-tip clearance or blade-tip timing in turbines, reflective intensity-modulated optical fiber sensors overcome several traditional limitations of capacitive, inductive or discharging probe sensors. This paper presents the signals and results corresponding to the third stage of a multistage turbine rig, obtained from a transonic wind-tunnel test. The probe is based on a trifurcated bundle of optical fibers that is mounted on the turbine casing. To eliminate the influence of light source intensity variations and blade surface reflectivity, the sensing principle is based on the quotient of the voltages obtained from the two receiving bundle legs. A discrepancy lower than 3% with respect to a commercial sensor was observed in tip clearance measurements. Regarding tip timing measurements, the travel wave spectrum was obtained, which provides the average vibration amplitude for all blades at a particular nodal diameter. With this approach, both blade-tip timing and tip clearance measurements can be carried out simultaneously. The results obtained on the test turbine rig demonstrate the suitability and reliability of the type of sensor used, and suggest the possibility of performing these measurements in real turbines under real working conditions.

## 1. Introduction

Blade-tip timing (BTT) and tip clearance (TC) are two critical parameters for turbine engineering, since their measurement and optimization lead to more effective, secure and reliable engines [1]. BTT is a technique for blade vibration measurements that uses the differences between real and theoretical blade arrival times to calculate the vibration amplitude of the blade. Pioneering works in the BTT technique were performed in the 70 s and during the last forty years ample research has been developed and published related to this technique. Some examples of these works, chronologically ordered, can be found in References [2–11]. Blade vibration measurements are crucial to assess turbine operation and predict blade failures due to fatigue [12]. Blade vibrations can occur due to different causes. For instance, combustors or stators can produce synchronous responses in the rotor blades, or even irregularities in the casing or in the intake geometry can produce non-regular pressure distributions that lead to synchronous responses in the rotor blades. In contrast, rotating stall or adverse flow-blade interaction with negative aero-damping can cause non-synchronous responses, such as flutter, which consists of a self-sustained aerodynamic instability. In order to predict the lifetime of blades and to prevent damages that can lead to huge repairing costs or even to engine destruction, a low-cost and effective blade vibration system is needed. The BTT technique fulfils both requirements detecting all blade vibrations.

Blade vibrations are usually measured with strain gauges. These devices can be used provided that the influence of their mass is negligible. Results are provided only for the blades on which gauges are mounted and the signals from the gauges have to be transmitted by telemetry or a slip ring. Despite their proven suitability, strain gauges require considerable instrumentation, their use is restricted to a few blades of the turbine and they are in physical contact with the blades. A second standard method is the frequency modulated grid system [13]. This method is based on small permanent magnets attached to the tips of some blades that induce an electromagnetic voltage in a wire installed in the casing. Its disadvantages are similar to those of the strain gauges [14].

As to the TC, it is defined as the distance between the blade-tip and the engine casing, and its usual values are 2–3 mm. This distance is one of the factors on which engine efficiency depends, as the latter increases as TC decreases. A high TC allows an amount of air to flow without generating a useful work, whereas a lack of clearance can put engine integrity at risk. A 0.25-mm-TC reduction is equivalent to a reduction of 1% in specific fuel consumption, and of 10 °C in exhaust gas temperature [15]. As a consequence, the engine works at lower temperatures and the life cycle of its components is increased. In addition to the economical benefit, aircraft noise and emissions are also reduced, implying additional environmental advantages.

For the measurement of TC, traditional methods make use of capacitive, eddy current and discharging probe sensors. Capacitive sensors are simple and inexpensive but they have a poor frequency response and require iron blades. Eddy current sensors provide non-contact measurements, but the magnetic disturbance of the turbine engine can interfere with their output. In addition, they need to be calibrated in advance, because they are highly dependent on the tip shape and temperature [16]. Finally, discharging probes, in the same way as eddy current sensors, require conducting blades and they only measure the shortest clearance. On the contrary, optical fiber sensors provide small size and simplicity, non-contact measurements and simple instrumentation, high sensitivity, resolution and bandwidth, insensitivity to electromagnetic interference and measurement of every blade [17]. In this paper we will show the results obtained for the BTT and TC measurement using an optical fiber bundle sensor.

## 2. Experimental Section

**Figure 1.**
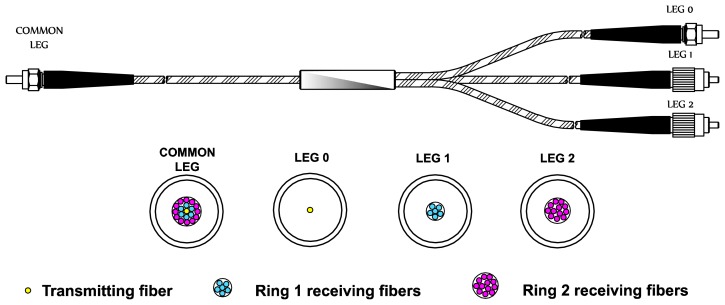
Trifurcated optical fiber bundle and legs cross-section.

All tests and measurements took place in the Aeronautical Technologies Center (CTA) facilities and were performed in a turbine rig with a rotor of 146 blades. The main component of the sensor is a trifurcated optical fiber bundle (manufactured *ad-hoc* by Fiberguide Industries, Stirling, NJ, USA, according to our requirements). The bundle structure is depicted in Figure 1: its length is 3 m and it consists of multimode glass optical fibers. Optical fibers temperature operating range goes from −190 to 350 °C, whereas the maximum temperature reached during the measurements is 88.1 °C.

**Figure 2.**
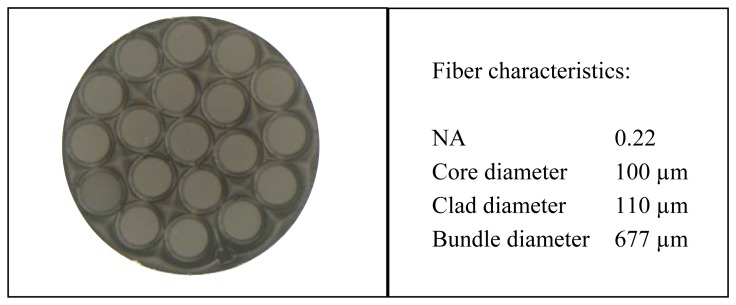
Microscope image of the cross-section of the common leg.

Figure 2 shows a microscope image of the cross-section of the common leg. The central fiber is the transmitting fiber which guides the light from the laser to the probe end in order to illuminate the blade. The reflected light is collected by two receiving fiber rings around the transmitting fiber. The inner ring is formed by six fibers that are gathered in leg 1 and the outer ring is composed of twelve fibers gathered in leg 2.

A trifurcated bundle is chosen to eliminate the effects of variations in the light source, reflectivity of the blade surface, and optical losses and misalignments between the probe and the target surface [16,18]. This is possible because the distance to the target is obtained as a function of the quotient of two photodetector voltages (V_1_ and V_2_), so the influence of any previous disturbance is cancelled. To evaluate the reflected light irradiance collected by each ring of receiving fibers, let us call *I*_0_ the light irradiance leaving the transmitting fiber in the common leg of the bundle. Then the optical irradiance at the end of legs 1 and 2 can be expressed as:

(1) $$I_{1}=K_{0}{\text{RI}}_{0}K_{1}F_{1}(d)$$

(2) $$I_{2}=K_{0}{\text{RI}}_{0}K_{2}F_{2}(d)$$

where *R* represents the reflectivity of the blade, the coefficients *K*_1_ and *K*_2_ account for the losses in the corresponding receiving fibers, each function *F*_1_(*d*) and *F*_2_(*d*) represents the relationship between the collected irradiance and the target distance for each group of fibers considered as a bifurcated bundle [19–24], and *K*_0_ is a factor that accounts for laser fluctuations.

Dividing both equations we obtain:

(3) $$I_{2}/I_{1}=K_{2}F_{2}(d)/K_{1}F_{1}(d)=\text{KF}(d)$$

Therefore, the quotient of the irradiances only depends on a constant related to the losses in the optical fibers and it is a function of the distance to the illuminated blade.

**Figure 3.**
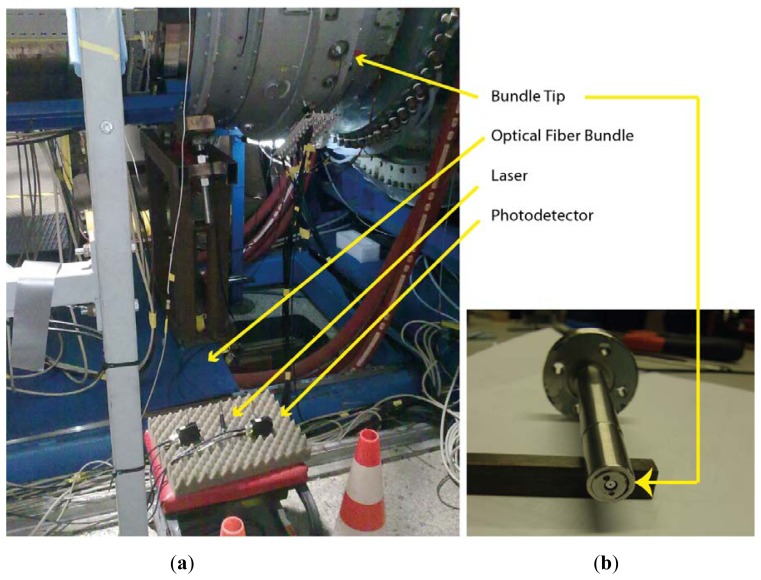
(**a**) Experimental setup for the tip clearance and tip timing measurements in the turbine rig at the CTA facilities. (**b**) Coupler to fix the bundle tip to the turbine casing.

We employ a 655-nm-wavelength laser (FP-65 7FE-SMA, Laser Components, Olching, Germany) as the light source with a typical power of 7 mW. The laser is coupled to leg 0. Two identical photodetectors (PDA100A-EC, Thorlabs, Dachau, Germany) are connected to the end of legs 1 and 2, which convert the reflected light collected by each ring into a voltage signal. The photodetectors consist of a reverse-biased PIN photodiode and a switchable gain transimpedance amplifier. The adjustable gain range goes from 0 to 70 dB, and the bandwidth decreases from 1.5 MHz to 2 kHz as the gain increases. The maximum output signal amplitude for a 50 Ω load is 5 V through a BNC connector. To acquire the output signal an Agilent Technologies (Santa Clara, CA, USA) Infinium MSO9104A oscilloscope was used. It has four analog channels of 1 GHz and a maximum sample rate of 20 Gsa/s. All these components are depicted in Figure 3(a) with the exception of the oscilloscope, which was placed at the control room due to the high temperatures reached. In order to carry the signal from the photodetectors to the oscilloscope and reduce the noise of the engine, two 25 m long double-shielded coaxial cables were used.

The first problem we encountered during the assembly was how to couple the common leg SMA connector to the casing of the turbine. It was solved using a threaded tip in order to fix the SMA connector to a coupler which was inserted in a probe hole, shown in Figure 3(b), that placed the bundle tip at a distance of 0.45 mm from the inner face of the casing.

**Figure 4.**
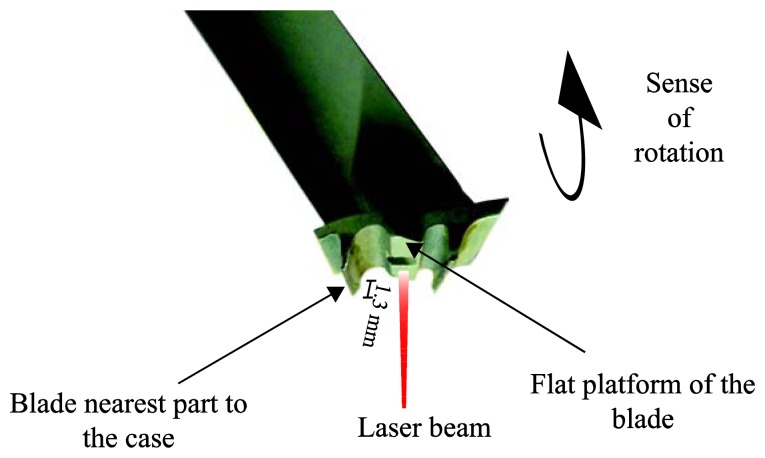
Blade profile image.

In Figure 4 the blade shape can be observed. We decided to illuminate the flat platform of the blade, so that the whole laser spot illuminates a flat surface and, therefore, most of the reflected light returns to the bundle. Since the difference between the flat platform and the nearest part of the blade to the casing is 1.3 mm, 1.75 mm should be subtracted from the measured distance to obtain the real TC.

**Figure 5.**
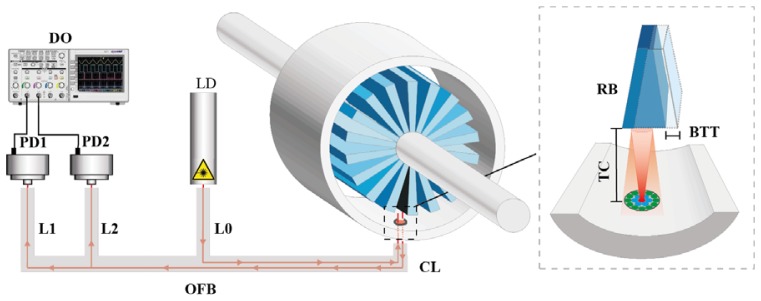
Experimental set-up and operational principle. Legend: DO: Digital Oscilloscope; PD1: Photodetector 1; PD2: Photodetector 2; OFB: Optical Fiber Bundle; LD: Laser Diode; TC: Tip Clearance; BTT: Blade Tip Timing; RB: Rotor Blade; L0: Leg 0; L1: Leg 1; L2: Leg 2; CL: Common Leg.

The experimental set-up is depicted in Figure 5. In addition to the sensor components, the inset shows the operational principle of the sensor. The light from the illuminating fiber is reflected by the blade and collected by the two rings of receiving fibers. Afterwards, the photodetectors connecting to each receiving leg of the optical fiber bundle carry out the photoelectric conversion. Finally, the oscilloscope acquires and saves the signals from both photodetectors. Additionally, the inset clarifies the tip timing and TC parameters.

**Figure 6.**
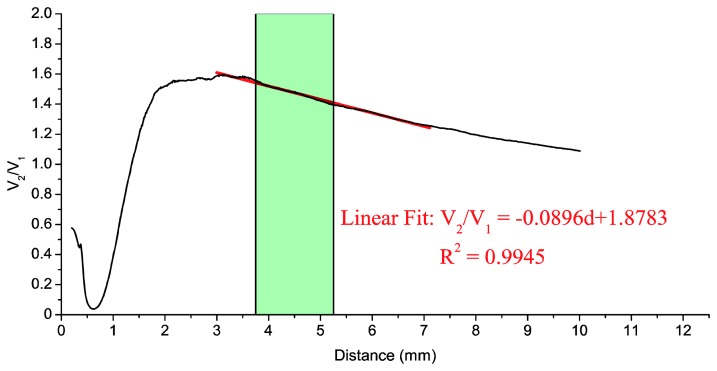
Calibration curve of the optical fiber sensor: quotient of photodetector voltages (*V*_2_/*V*_1_) as a function of the blade distance (*d*). The red line represents the fit to the calibration curve of a straight line in the range 3–7 mm. The shaded area corresponds to usual values of the TC (without correction).

During the calibration we used the same components except for the oscilloscope, which was replaced by two digital multimeters (notice that during the calibration process a high acquisition rate is not necessary and that multimeters are much easier to automate). The bundle tip was fixed to a self-made SMA adapter. The blade was installed on a linear stage and it was longitudinally moved away from the common leg of the bundle in 10-μm steps. Using this procedure we obtained the calibration curve of the sensor. This calibration was carried out both in darkness and in the presence of light, and the differences between them were found negligible. The calibration curve is shown in Figure 6.

Taking into account that typical TC values for our turbine range between 2 and 3.5 mm, and that we have to add the 1.75 mm offset distance, the measurement range should be between 3.75 mm and 5.25 mm (represented by the shaded area). A straight line has been fitted to the calibration curve, as shown in Figure 6, by the least-square method for the 3–7 mm interval, with a coefficient of determination *R*^2^ = 0.9945.

## 3. Results and Discussion

**Figure 7.**
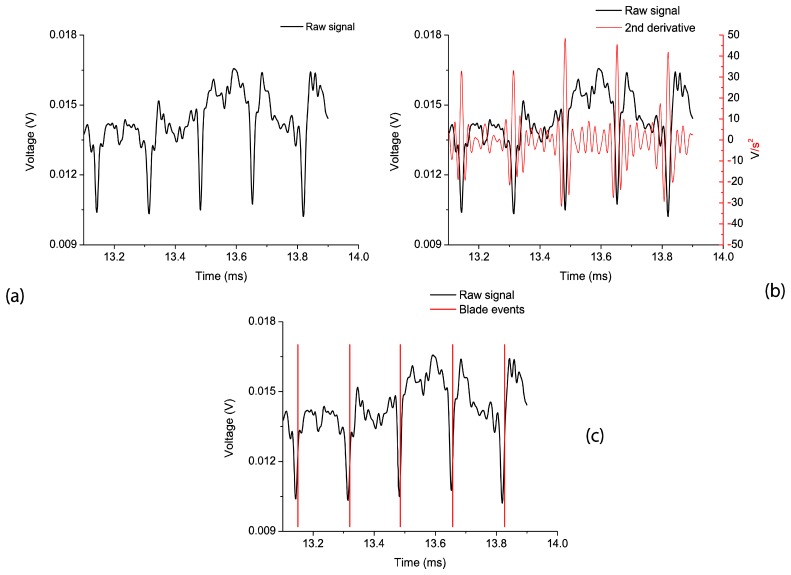
(**a**) Raw signal of photodetector 2. (**b**) Second derivative of the raw signal. (**c**) Detected blade events.

An important restriction of our acquisition system is that the oscilloscope has only 8 bits for vertical resolution. Therefore, we performed an oversampled acquisition to obtain a better-detailed waveform. For the case of TC measurements the sampling frequency was 250 Msa/s, and the oscilloscope memory depth allowed a maximum acquisition time of 82 ms from both photodetectors. In BTT measurements the accuracy in the amplitude of the signals is not as critical as in the former case, so the sampling frequency was set to 250 Ksa/s, obtaining 82 s of acquisition time. Both measurements were performed for 84 different working conditions (working points) of the engine. To identify each revolution, a blade with a particular reflection pattern has been used. The data from the sensor are post-processed after a low-pass filtering (cut-off frequency 50 KHz). Once the sensing principle is validated, a real time implementation will be possible. In Figure 7(a), we can observe the post-filtering signal from photodetector 2 for a specific working point where the turbine rotates at 2,400 rpm.

### 3.1. Tip Timing Measurements

The BTT technique is based on the measurement of the arrival times of all the blades. If the blades do not vibrate, the theoretical arrival times are obtained from a simple relationship involving the number of blades, the rotation frequency and the blade radius. However, if the blades do vibrate, their arrival times precede or succeed the theoretical non-vibrating arrival times. The difference between the theoretical and the real arrival time is related to blade deflections and it is used in the post-processing section of the system [25].

Furthermore, by placing probes at different circumferential positions, a sine wave model can be fitted to the blade deflections measured at each position. From these sinusoidal fits, we will be able to obtain the frequencies and amplitudes of the vibrations.

In our tests, a single probe was used to measure the blade arrival times. With this single probe application it is not possible to carry out an appropriate model fitting and a complete modal analysis cannot be achieved. Nevertheless, it is still possible to perform a travelling wave analysis in order to obtain the average amplitude of the blade tips at a particular nodal diameter (*ND*) (note that the minimum value of *ND* is 0, which is obtained when all the blades vibrate with the same phase, whereas *ND* reaches its maximum value (half of the number of blades) when each blade is out of phase with its neighbour). This travelling wave analysis can be used for monitoring the integrity of the blades against flutter, crack propagation or foreign object damage (*FOD*).

The travelling wave mode is the vibration condition of the blades. In a bladed disk system, the blades vibrate at the same amplitude but with a phase lag between them. This is known as the inter blade phase angle (*Ibpa*). This phase lag between the blades is related to the nodal diameter (*ND*) of the bladed disk mode according to:

(4) $$\text{ND}=\frac{2\pi n}{\text{Ibpa}}$$

where *n* is the number of blades.

As a consequence the frequency detected by the probe (*f_probe_*) is the frequency of the blade (*f_blade_*) plus the nodal diameter multiplied by the rotation frequency (*ω*), *i.e.*, [26]:

(5) $$f_{\text{probe}}=f_{\text{blade}}+\omega \text{ND}$$

Let us define the engine order (*EO*) as:

(6) $$\text{EO}=\frac{f_{\text{blade}}}{\omega }$$

Dividing Equation (5) by the rotation frequency and substituting Equation (6):

(7) $$f_{\text{probe}}/\omega =f_{\text{blade}}/\omega +\text{ND}=\text{EO}+\text{ND}$$

Therefore, a probe placed in the casing that measures the frequency of a rotating part detects not only the frequency of the blade, but also the phase lag that a blade has with its neighbour, so that it is not possible to discriminate *EO* from *ND*, because the probe detects the arrival time of each blade as a superposition of both terms.

The travelling wave analysis is mainly used for non-synchronous responses, such as flutter, where *EO* + *ND* has non-zero values. For synchronous responses it turns out that *EO* + *ND* = 0, since the excitation and the response are in phase and, therefore, synchronous responses are more difficult to detect with the travelling wave analysis.

Finally, the working point of the turbine is defined by two parameters: the rotational velocity *N* and the work per unit mass flow *Ws*. The specific work is defined as [27]:

(8) $$\text{Ws}=C_{p}*\Delta T_{0}$$

where *C_p_* is the air specific heat capacity at a constant pressure and *ΔT_0_* is the total temperature drop within the stage.

Figure 7(a) shows the raw signal obtained with the optical probe for a test performed at nominal working conditions (2,400 rpm). Notice that we can estimate the arrival time of each blade by calculating the second derivative of the signal. This derivative gives the change in concavity/convexity of the raw signal as can be seen in Figure 7(b). Choosing a threshold value for the second derivative of the raw signal, the blade arrival event can be obtained for every blade. In Figure 7(c), the raw signal together with the blade arrival events can be observed.

**Figure 8.**
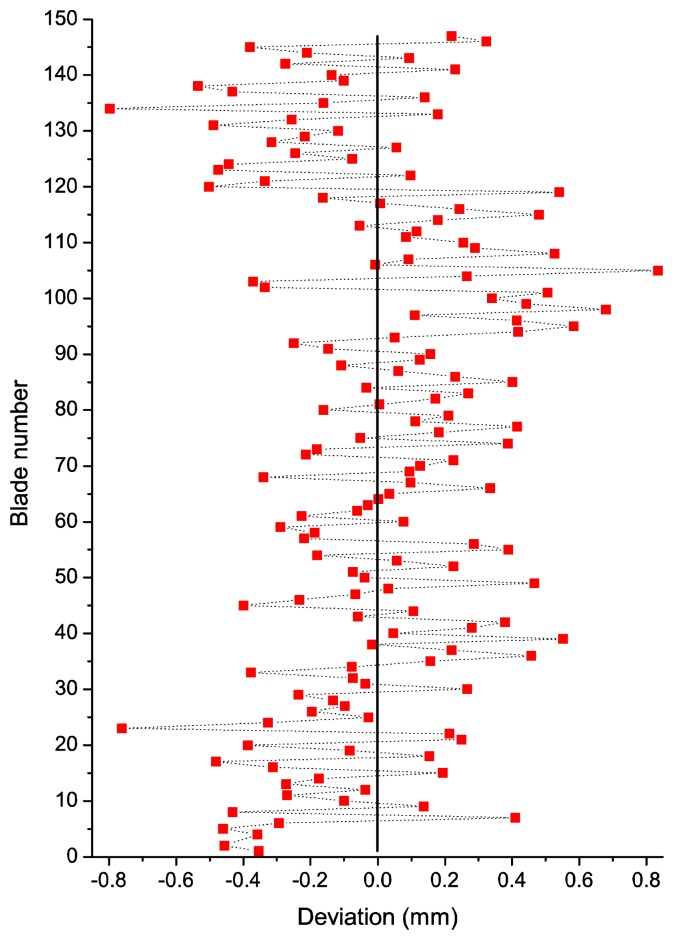
Deviation of each blade from the equilibrium position in a complete revolution.

From the arrival times we can obtain the deflection or deviation of each blade from its theoretical equilibrium position. The deviations from the theoretical equilibrium position in one revolution for every blade are shown in Figure 8. This deviation provides us with useful information for health monitoring to predict possible damages in blades. By plotting the deviations in real time, flutter or crack propagations can be detected as the deviation of a certain blade increases in time and gets close to predefined pre-alarm values.

**Figure 9.**
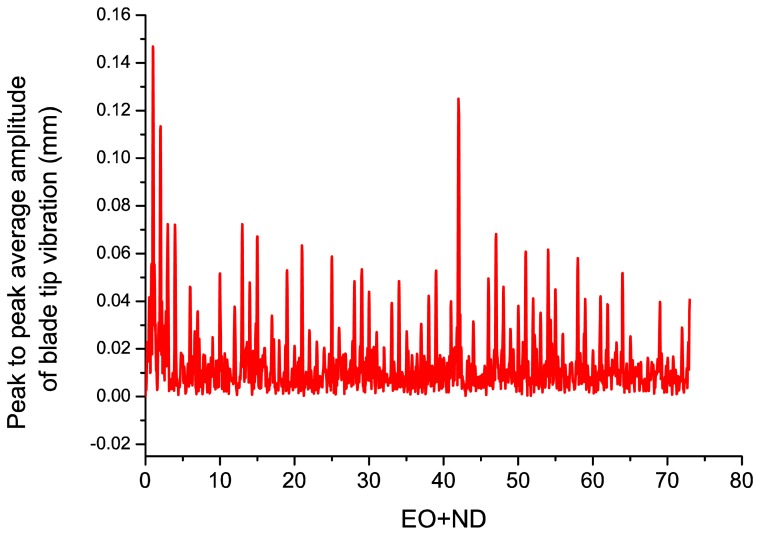
Travelling wave frequency spectrum of the system.

With the deflection values of the blades a fast Fourier transform can be performed to obtain the travelling wave spectrum of the system, which gives the average tip amplitude of all the blades, as shown in Figure 9. The travelling wave spectrum gives an average value of all the blade vibration amplitudes at a certain nodal diameter and it can also be used to monitor the system in real time, in order to check that the average blade tip amplitude does not exceed a predefined maximum value.

Figure 9 depicts the peak to peak average amplitude of blade tip vibration. There is a dominant peak of 0.15 mm at *EO* + *ND* = 1. This is probably a solid rigid rotation (*EO* = 1, *ND* = 0) due to some unbalancing of the rotor or the facility instead of a blade vibration mode.

There is another important peak at *EO* + *ND* = 42 of 0.13 mm that could be due to a low amplitude non-synchronous response, such as a flutter. When a blade starts moving, the surrounding flow exerts an aerodynamic force on it, and the direction and phase of this force can dampen or speed up the motion of the blade, leading to flutter. The *Ibpa* determines the phase between the local unsteady flow and local blade motion and this phase affects the unsteady aerodynamic work done on the blades. Unfavorable phase angles can lead to positive work being performed on the blades, which results in flutter.

All in all, the vibration amplitudes are always below 0.2 mm in the frequency spectrum of Figure 9 which is indicative of the stability of the rotor. More tests were performed for different rotation frequencies, leading to similar conclusions.

In order to discriminate the frequency of the blade and the nodal diameter, more probes should be placed in the casing in other circumferential positions. In the future, more probes are planned to be mounted in order to perform more detailed modal analysis of the rotors.

### 3.2. Tip Clearance Measurements

**Figure 10.**
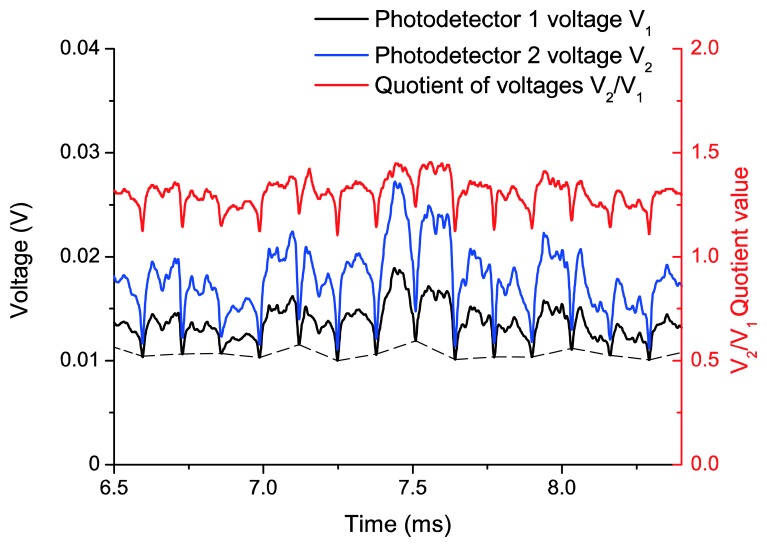
Filtered signals from the photodetectors and their quotient at a working point of 3,148 rpm.

As we have already mentioned, TC represents the gap between the blade tip and the engine casing. This distance is obtained as a function of the quotient of two photodetector voltages, and the calibration curve of Figure 6 is used to relate both quantities. Figure 10 shows the two filtered photodetector signals and its quotient during the acquisition of a working point at 3,148 rpm. In this figure, 15 blades can be observed. If we pay attention to the minimum values that limit each blade, we can notice that each minimum has a different level (dashed line in Figure 10). This offset is probably due to the vibrations of the rotor-shaft assembly, so the first step in the signal processing, after the low pass filtering, is to remove this offset so that the signals start at the same level for all blades. Afterwards *V*_2_ and *V*_1_ are divided and its maximum value is found for a complete revolution. By definition, the TC is the distance corresponding to the maximum value of the quotient.

As we have already mentioned, 84 different working points were acquired during turbine assessment. Since the TC value depends mainly on the number of rpm, for a first evaluation of the sensor operation, all working points with the same rpm were averaged to obtain a TC for each turning speed. The optical sensor response was compared with a discharging probe sensor (Rotatip RCSM4 from Rotadata, Derby, UK) used by CTA. After appropriate data processing, the results for both sensors are shown in Table 1.

Whereas the optical sensor can obtain the distance to the casing for each blade at any instant, the discharging probe sensor gets only the smallest clearance of all the blades in an unknown revolution. The measurement instant is, therefore, not the same for both sensors, and even though measurements are taken in a steady state, they are measuring different distances (different vibration modes of the blades in different revolutions). It must be also taken into account that the standard deviation in the data from the sensor before speckle correction was about 150 μm. However, since our aim is to compare the behaviour of both sensors, we believe it is useful to show the obtained results. The latest statement is demonstrated by the fact that the differences between the two sensors are on the order of some tens of microns and the relative difference is less than 3%, as it can be observed in Table 1.

## 4. Conclusions

An optical sensor based on a trifurcated optical fiber bundle has been used to carry out BTT and TC measurements using a reflected intensity-modulation technique. Despite the limitation of using a unique probe for BTT measurements, the travelling wave spectrum has been obtained and with it the average vibration amplitude for all blades. With respect to TC measurements, a good approximation to a commercial sensor has been achieved, with differences lower than 3% for average TC values at each turning speed.

The great potential of optical fiber bundles for this kind of measurements has been demonstrated. The main novelties of this work with respect the previous ones are the possibility to simultaneously carry out both BTT and TC measurements with the same probe, and the reporting of these results as obtained from a real test of a turbine rig. In addition, the measurement system is a non-contact one, which allows to obtain information from all the blades with very short instrumentation times and relatively low cost. In future tests, several probes will be installed in the casing to make the most of the BTT measurements.

## Figures and Tables

**Table 1.** Tip clearance measurements obtained during turbine tests.

**Working Point (rpm)**	**Discharging Probe Tip Clearance (mm)**	**Optical Sensor Tip Clearance (mm)**	**Difference (%)**
3148.52	2.890	2.954	2.22
3390.71	2.919	2.961	1.42
3632.91	2.893	2.843	1.72
3875.10	2.851	2.840	0.38
